# Staff experiences within the implementation of computer-based nursing records in residential aged care facilities: a systematic review and synthesis of qualitative research

**DOI:** 10.1186/1472-6947-14-54

**Published:** 2014-06-20

**Authors:** Anne Meißner, Wilfried Schnepp

**Affiliations:** 1 Department of Nursing Science, University Witten/Herdecke, Witten, Germany

**Keywords:** Electronic health record, Health information technology, Computer, Technology adoption, Satisfaction, Long-term-care, Benefit and costs, Cost benefit analysis, Net benefit, Information system success, Attitude

## Abstract

**Background:**

Since the introduction of electronic nursing documentation systems, its implementation in recent years has increased rapidly in Germany. The objectives of such systems are to save time, to improve information handling and to improve quality. To integrate IT in the daily working processes, the employee is the pivotal element. Therefore it is important to understand nurses’ experience with IT implementation. At present the literature shows a lack of understanding exploring staff experiences within the implementation process.

**Methods:**

A systematic review and meta-ethnographic synthesis of primary studies using qualitative methods was conducted in PubMed, CINAHL, and Cochrane. It adheres to the principles of the PRISMA statement. The studies were original, peer-reviewed articles from 2000 to 2013, focusing on computer-based nursing documentation in Residential Aged Care Facilities.

**Results:**

The use of IT requires a different form of information processing. Some experience this new form of information processing as a benefit while others do not. The latter find it more difficult to enter data and this result in poor clinical documentation. Improvement in the quality of residents’ records leads to an overall improvement in the quality of care. However, if the quality of those records is poor, some residents do not receive the necessary care. Furthermore, the length of time necessary to complete the documentation is a prominent theme within that process. Those who are more efficient with the electronic documentation demonstrate improved time management. For those who are less efficient with electronic documentation the information processing is perceived as time consuming. Normally, it is possible to experience benefits when using IT, but this depends on either promoting or hindering factors, e.g. ease of use and ability to use it, equipment availability and technical functionality, as well as attitude.

**Conclusions:**

In summary, the findings showed that members of staff experience IT as a benefit when it simplifies their daily working routines and as a burden when it complicates their working processes. Whether IT complicates or simplifies their routines depends on influencing factors. The line between benefit and burden is semipermeable. The experiences differ according to duties and responsibilities.

## Background

Nursing documentation is recognized as a necessity in professional nursing. Until the last century, paper-based documentation systems were those most commonly used in Germany.

Nursing has become more complex, the amount of documentation has increased immensely. Almost all companies are searching for solutions that reduce the effort associated with documentation and at the same time offer a professional and appropriate documentation product. IT-based nursing documentation may be one possible solution [1-3].

During recent years the introduction of electronic nursing documentation systems in nursing homes in Germany has increased rapidly [4-9]. According to a recent non-representative study, 43.5% of the facilities for the elderly in Germany already use a computerized system. Another 11.3 % of Residential Aged Care Facilities (RACFs) plan to implement a computer-based system [10]. Statistically representative data for Germany is currently not available.

The change to computer-based nursing records is associated with capital asset and resource management costs. RACFs implemented IT-based nursing documentation realizing more benefits with the change than effort and cost associated with the change [3,11]. It should be emphasized that the added value will never be achieved by the IT itself, but always through a process optimization achieved by the IT [6-9].

For example, a) time efficiency resulting from the improvement of documentation quality is a deciding factor for the implementation of electronic documentation systems in hospitals [12] and nursing homes [3,13]. In another example b) quality improvements as well as c) better information processing are also key factors for implementing such systems [3]. The above noted aspects can be summarized as process optimization.

However, as of today, there is no empirical evidence that electronic nursing documentation systems add value to nursing, such as a) improved time management or b) improving information handling or c) increasing quality, the latter split in c1) quality of documentation (factual and professionally correct, continuous, complete) and c2) quality of care (more safety and better quality of life for the patient) [14,15]. Finally, consensus exists that those objectives could be achieved with the full implementation of IT. Nevertheless, at present, there is no evidence that the relevant objectives will actually be achieved and ‘full’ implementation is not clearly defined.

At this point, it must be stated that full implementation includes not only completely paperless records, but is also recognized as computer system success. Moreover and according to DeLone and McLean [16], user satisfaction is a key factor of computer system success and IT integration, and the impact a computer-based system has on a user’s job directly affects user satisfaction [16].

At present the literature shows a lack of understanding regarding staff experience in order to possible benefits when using computer-based documentation.

There is inadequate evidence concerning what must be done to ensure and maintain process optimization (benefit). Research indicates opposing trends [4,5,17-35].

Finally, to integrate IT in the daily working processes, the employee is the pivotal element. Therefore it is important to understand nurses’ experience with IT implementation. The need to carry out a synthesis for a deeper insight and understanding of the phenomena is given by the difficulty of translating knowledge from individual studies to practice and research.

## Methods

### Aim

The aim of this study is to explore staff experiences within the process of the implementation of computer-based nursing records. The following question guided the literature search:

– How does staff describe their experiences with the benefits observed with respect to the computer-based records and their daily work?

### Design

Data was analyzed and synthesized by using the meta-ethnographic approach from Noblit and Hare [36]. The core of the meta-ethnographic approach is the reciprocal translation meaning, “in an iterative fashion, each study is translated into the terms (metaphors) of the others and vice versa [36]: 38”. This method encourages the researcher to understand and transfer ideas, concepts and metaphors across different studies.

### Search strategy and sample

The development of IT is progressing rapidly. In addition, the millennium change in 2000 (“Y2K”) and the introduction of the Euro in Europe have led to major changes in the IT industry. The literature search was therefore limited to the period 01.01.2000 - 01.01.2013.

To ensure that all relevant literature was included, the first step consisted of a search of studies concerning computer-based nursing documentation in general.

**Figure 1:**
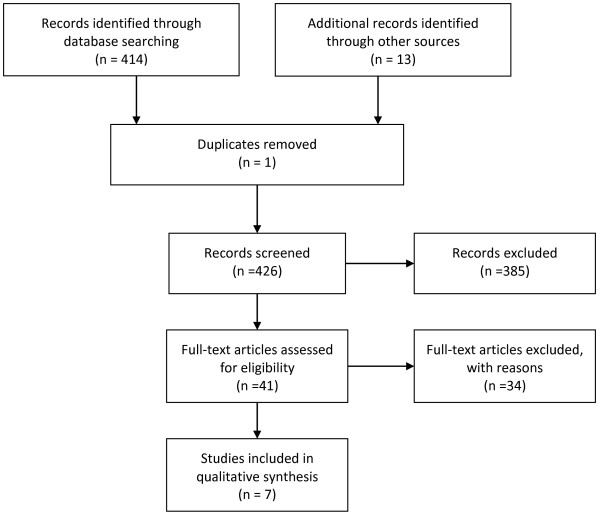
PRISMA Flowchart of search.

Therefore a search was carried out in PubMed, CINAHL and Cochrane. The following search terms were used and linked with AND and OR: nursing documentation*, nursing record*, nursing information system*, electronic*, computer*, technolog*, nursing home*, resident*, long-term.Following further reading of abstracts, only those studies were chosen that a) address computer-based nursing records in RACFs, b) have been published in the English or German language, and c) used a qualitative design. To ensure that only studies that fulfill research quality criteria were included, only those were chosen that d) had been published in a peer-reviewed journal. Next, all remaining articles were read in full text and all studies excluded that did not meet the inclusion criteria. In addition, a manual search was conducted from the reference lists of the articles obtained. For search details see Figure 1.

Studies that referred to the setting “hospital” were excluded. Furthermore, studies were excluded that focused on standardized techniques, e.g. nursing languages or Minimum Data Set. Those studies were excluded because the focus of such studies is on the technique rather than on the IT system itself.

This systematic review adheres to the principles of the PRISMA statement [37].

Accordingly, after reviewing the full text, seven articles were selected. For details of the selected articles see Table 1.

**Table 1:** Summary of analyzed articles

**Authors**	**Aims and objectives**	**Methods design**	**Interview**	**Focus group**	**Observation**	**Results**
		**Data analysis**				
		**Sample**				
**Date of publication**						
**Country**						
**Title**						
Alexander et al. (2007) U.S.A. Clinical information systems in nursing homes: an evaluation of initial implementation strategies [38]	To explore implementation strategies, employee experiences, and factors influencing employee satisfaction	Explorative 4 nursing homes, 6 months after implementation	23	22	---	Five themes emerged: (1) perception and cognition, (2) change, (3) workable system, (4) competence and (5) connectedness.
		Focus groups (22 à 60 Min.)				
						Implementation strategies associated with lower satisfaction were availability of equipment, training resources, and the presence of professional information technology. The experiences differ [according] to the role.
		Unstructured observations (< 5 min., made when using the technology, (n=?) semi-structured interviews (unknown type) (n=23) axial coding				
Cherry et al. (2008) U.S.A. Factors affecting electronic health record adoption in long-term care facilities [39]	To gain information about Long Term Care leaders’ general understanding about Electronic Health Records (EHR) and identify factors that hinder and facilitate EHR in Long Term Care	Explorative	---	34	---	Primary barriers identified were costs, the need for training and the culture of change. Primary facilitators were training programs, well-defined implementation plans, evidence that the electronic systems will improve care outcomes.
		Focus groups (34) via telephone conference call with directors of nursing, Administrators and corporate executives divided into users and non-users				
Cherry et al. U.S.A. (2011) Experiences with electronic health records: early adopters in long-term care facilities [40]	Providing a description of the early users’ experiences, challenges and benefits with Electronic Health Records in Long Term Care	Explorative	70	---	10	The RACF employees who work with EHR systems on a daily basis were positive about their experiences. In particular, operational improvements were achieved through increased access to resident information, cost avoidance, increased documentation accuracy and implementation of evidence-based practices.
		Semi-structured interviews of unknown type, group-observation				
		10 "freestanding" Sites, one-site visit for 6-8 hours per visit with the following schedule for the face-to-face interviews: (a) 60 min for facility tour, (b) 45 min with the administrator, (c) 45 min with the DON, (d) 45 min with a group of assistant DONs and charge nurses, (e) 45 min with a group of direct care staff, (f) 45 min with residents and family members, (g) 60 min for observation on the unit during shift change				
Munyisia et al. (2012) Australia The impact of an electronic nursing documentation system on efficiency of documentation by caregivers in a residential aged care facility [26]	To examine the effect of the introduction of an Electronic Health Records system on the efficiency in a Long Term Care facility	NOT INCLUDED IN THIS REVIEW:	8	---	---	Qualitative interviews to gain a better understanding 1. Personal Carers were happy in general because of quicker access and release from referring to written doctors notes
		Longitudinal cohort study				
		INCLUDED IN THIS REVIEW:				
						2. Certain information items were double charted (Paper and EHR) due to organizational reasons
		Explorative semi-structured Interviews (n=8) unknown type 6 and 12 months after introduction				
						3. It took longer to complete some documentation tasks using a computer (too many clicks to enter data)

		Qualitative content analysis				4. Continuous training is needed for some caregivers to effectively use the EHR

Rantz et al. (2011) U.S.A. The use of a bedside electronic medical record to improve quality of care in nursing facilities: a qualitative analysis [41]	To examine the effect of the introduction of a bedside electronic medical record on the improvement of care in nursing facilities	(Part of the study of Alexander et al.)	120	22	?	Communication and information was improved which led to a general improvement of patient care
		Explorative qualitative interviews (n=120), observations (?), focus groups (22) content analysisin all 4 homes 6,12, 18 months after implementation, additional interviews took place (n=?) 24 months after implementation in 2 homes				
						Experience of limited time due to EHR (Direct Carer) vs. saved time (Management)
						Too much time for operating and managing the system
Yu et al. (2008) Australia Caregivers' acceptance of electronic documentation in nursing homes [35]	The aim of the study was to investigate nursing home caregivers' acceptance of electronic documentation	NOT INCLUDED IN THIS REVIEW	12	---	---	Some staff (4) with low experience wished for more time in the beginning and more instructions
						Some staff (4) often used computers at home felt the software was easy to use
		Questionnaire survey				Other staff (4) felt they needed more practice than theoretical lessons
		INCLUDED IN THIS REVIEW				
		Semi-structured interviews unknown type after 11 weeks computer-based (n = 12)				
		Paper-based n =?				
		One Home that implemented an Electronic Health Records; one home remained paper-based.				
Zhang (2012) Australia The benefit of introducing electronic health records in residential aged care facilities: A multiple case study [42]	The aim of this study was to identify the benefits of Electronic Health Record in Long Term Care and to examine how the benefit have been achieved	Explorative semi-structured Interviews (n=110) content analysis, theoretical sampling	110	---	---	BENEFITS TO THE STAFF
						Convenience and efficiency in data entry, distribution, storage and retrieval
						Ease of access more information to better understand the residents, the service and peer-learning
						Empowering care staff
						BENEFITS TO THE RESIDENTS
						Improving Quality of Care
						BENEFITS TO THE RACFs
						better information management
						Improving the communication system
						Improving access to funding facilitating care quality control better work environment educational benefits

**Data Foundation (at least) // 23 Interviews and 22 focus groups removed due to doubling**			**320**	**56**	**10**	

### Analysis & synthesis

Noblit and Hare [36] defined a seven-step procedure for guiding a meta-ethnographic approach (Table 2).

**Table 2:** Seven Phases of Noblit and Hare’s meta-Ethnography

1. Phase	Getting started
2. Phase	Deciding what is relevant to the initial interest
3. Phase	Reading the studies
4. Phase	Determining how the studies are related
5. Phase	Translating the studies into one another
6. Phase	Synthesizing translations
7. Phase	Expressing the synthesis

#### Phase 1: Getting started

According to Noblit & Hare [36], ‘getting started’ includes defining a research interest that qualitative research might enlighten. In our case the motivation for synthesizing the body of qualitative evidence is mostly based on the work of Ammenwerth et al. [43] and Urquhart et al. [15]. The authors stated that quantitative methods might not be sufficient to explore why individual wards react differently to computer-based nursing documentation.

#### Phase 2: Deciding what is relevant to the initial interest

This next phase involves several decisions on ‘what is relevant’. The rationale for search strategy, inclusion and exclusion criteria is presented in the section ‘search strategy and sample’.

#### Phase 3: Reading the studies

Even Noblit and Hare [36] in their original work state that this phase is not particularly clear. They interpret this phase as repeated reading with extensive attention to the details of each study. We understand this to mean that we should familiarize ourselves with the selected studies by reading them many times, mostly in full, but also in part.

#### Phase 4: Determining how the studies are related

To determine how the studies are related Noblit and Hare advocate forming a list of key metaphors, phrases, concepts and their relation to each other and juxtapose them.

In this study see Table 3 below for examples.

**Table 3:** Metaphors for technology problems

	**Alexander et al. **[38]	**Cherry et al. **[40]
Situation	*When part of the system did not readily interface*	*When the systems go down all work stops.*
Behavior	*it was viewed as a detriment.*	*Everyone seems lost without the computer.*

Italicized quotations represent the views of participants of included studies.

The data within each category formed the basis for the reciprocal translation described in the following.

#### Phase 5–7: Translating the studies into one another, synthesizing translations and expressing the synthesis

The syntheses cannot be reduced to a set of mechanistic tasks. When a concept arose, all the studies were searched for similar meanings (metaphors). We then built a line-of-argument synthesis, which interpreted relationships between the concepts. This section is merely an effort to express the synthesis. In practice, of course, the phases overlap.

The key concepts of each article are shown in Tables 4 and 5.

**Table 4:** Translation between studies: Possible benefits through the IT

	**Different information processing**	**Quality of documentation and resident care needs**	**Additional or lost time**
Alexander et al. [38]	Administrators were optimistic that this technology could improve management oversight and quality management	Administrator*s* were optimistic that this technology could improve documentation of resident care	Administrator:
			*nursing homes that implement [technology] need to be warned about the increased need for manpower during the initial months.*
	Frustration set in when expectations were not met. This increased staff suspicion and decreased desire to work with the system.		Frustration set in when expectations were not met, problems not solved in a timely manner
	Licensed nurses liked being able to view many things about resident care at once		
	liked being able to know what was done for their residents in real time identified increased documentation in comparison to the paper record		
	When the documentation system wasn’t working properly, staff stated they didn’t chart. Others indicated that backup systems for documentation were created. Concerns surfaced about increased potential for errors resulting from service duplication.		
Cherry et al. [39]	The user group suggested that supervisors were able to more easily monitor documentation of resident care activities, regulatory compliance issues, or staff education needs	They agreed that improvements in the quality and accuracy of documentation would be realized.	They agreed that improvements in the efficiency would be realized.
			Staff would spent less time in documentation tasks
		The user group suggested that supervisors were able to more (…) quickly identify resident care needs and address quality of care issues (…)	
	Specific aspects of care discussed included easier access to charts and medical information		
		Staff would spend more time in resident care	
		Better quality of care	
		Ability to provide automatic alerts (plausibility check)	
Cherry et al. [40]	Administrators:	Administrators:	DONs & Charge Nurses:
	Staff were able to provide better information because of immediate access	Better care to residents because of immediate access to computerized records	Nurse supervisors generally believed that the system allowed direct care staff to spent more time with residents and less time in documentation
	Immediate access to medical records allowed staff to access resident records without wasting time	Improved consistency, accuracy, and quality of documentation	*Gave the nurses more time on the floor since the paperwork went faster*
		*Fewer holes in documentation from a caregiver’s standpoint*	
	Direct Care Nurses:	DONs and Charge Nurses:	Direct Care Staff
	Nurses’ notes and notes by other caregivers are much easier to read	More consistent and legible documentation	About half the nurses reported that they had more time to spend with residents because of less time charting, and because of less time looking for “missing” charts, and about half reported no change or an increase in time required for charting and that they had less time with residents because of the amount of time spent in documentation activities Reports regarding the time required to admit a new resident was mixed, with some nurses reporting that new admissions were much easier and quicker and others reporting that it took much longer.
	Important issue discussed was the need for more information about the residents they care for	More thorough assessments with assessment templates that guide nurses through body systems for documentation and to help nurses improve observations skills	
	Ease of access to patient information was a definite benefit identified by the nursing staff	Direct Care Nurse:	
	Several noted how information on residents, including diagnosis and demographics, is now more readily available	Half reported Care Plans were easier to originate and maintain, half reported that it was more difficult	
	Missing charts didn’t matter because the information was in the computer	Improved documentation were definite benefits identified by the nursing staff	
	Information is more readily accessible	Quality of care was neutral (no change) to improve after the implementation	
	DON and Charge Nurse:	*Guided templates improve observation skills, which in turn provides for better care for the residents*	
	Ability to track and trend quality indicators	*We are able to more proactive address residents’ problems*	
	Increased ability to monitor staff and complete chart audits in very timely manner	Additional information increases a nurses’ awareness of the patient condition and allows for better care	
	Immediate access to records for any authorized staff member	More legible and accurate information	
Munyisia et al. [26]	The PCs were happy with the electronic documentation system because the access to the residents’ notes had been improved.	The paper-based record helped them make real-time care decisions	*I get a resident’s note on a computer at a finger click. Unlike using the manual system that required me to go over there (points a filing cabinet), search for a folder, come back, find the right page, and when the page was missing, go and get a photocopy. Therefore, access to one resident’s notes would probably take me 20 minutes before I sit down and start writing*
	*I get a resident’s note on a computer at a finger click.*		
			*When there was a clinic here (at the facility), the doctor wrote everything on the computer. Therefore I did not have to write progress notes because the doctor has already done it*
			*The only real problem I have is with the continence charts, it takes so long to enter everyone’s information in the system. It can take up to one hour to enter data and when using the paper system, it is just a 5 Min. job*
			*It does get slow to enter data into the computer that you eventually give up*
Rantz et al. [41]	Communication about resident care was reported as improved	Improvement with documentation was noted	All expressed concern that there was limited time to spent with residents and that the required documentation and time spent in managing the technology limited the amount of time actually spent with residents
	Easier access of information improved communication	Licensed and certified staff believed that the care was safer through the use of the system.	
	All stakeholders concluded that information was more easily accessible	Some licensed staff commented that the assessments caused them to think about what to assess and that it helped them identify problems that they might not have otherwise found.	
			The system required time to operate and manage.
			Documentation is too time consuming and a burden
			Documentation is perceived as too time consuming
			Frustration set in when the system don’t work (that causes more time)
Yu et al. [35]	---	---	---
Zhang et al. [42]	The most common viewed benefits for individual staff members are (…) more information to better understand the residents	Better understand the residents due to more information	The most common viewed benefits for individual staff members are (…) time efficiency
	More information to better understand the residents and the care services, to support peer learning and to facilitate performance appraisal for managers	Broader and more holistic view of the residents	Most of the staff saw reduction of paper work and time saving
	Easily check what care had been delivered	*For instance if I am unsure of how to do the palliative care, I can just easily click a button and find out it has been done for a similar patient at another facility*	*I think the computer is quicker because you can get to delete stuff and you can fix it*
	*Able to see if something has been identified, has somebody done something about It, if there is a gap and ensure that is corrected.*	*It does improve what you want to do because you get the whole picture, not just what’s happened on your shift. It does impact on how I deal with a resident*	*Care plans on paper are very time consuming, and the computer make it faster, since when it’s written once, you don’t need to write it again*
	*It helps me identify what is needed by the staff*	Improvement in the quality of residents’ records led to improvement in the quality of care	*Care plans on paper are very time consuming, and the computer make it faster, since when it’s written once, you don’t need to write it again*
	*Opportunities to, like I said, all the information that we need or help out with the students today. Like they wanted to know a little bit about all the resident’s conditions and stuff, so I just set them up on my system and they sat on there for a couple of hours and they really enjoyed it. Say a thing, they were able to find out everything they wanted to know about all the residents as well.*	Quick response to resident’s care needs	*It’s easier for the care staff members to entry data even if they are only typing with two fingers*
		Quicker and easier care decisions, the system has an impact on clinical judgment and decision making	
		Better care follow up	
	The most common viewed benefits for individual staff members are ease of access		
	Some reported it was easier and quicker		
	Some noted quick data distribution		
	*I just need to enter it into the computer and then that information is there for the staff to see. So it saves a log of time.*		
	Quick data retrieval was a well-recognized benefit. They found it was quicker and easier to find data		
	*Being able to scroll through and the way the notes are broken up into different categories where you can select whatever it is you are looking for and be done fairly quickly*		

Italicized quotations represent the views of participants of included studies. Non-italicized quotations represent views of authors of included studies.

CNA = Certified Nurse Assistant.

DON = Director of Nursing.

PC = Personal Carer.

**Table 5:** Translation between studies: Hindering or promoting aspects for experiencing benefits through the IT

	**Ease of use and ability to use it**	**Equipment availability and technical functionality**	**Attitude**
Alexander et al. [38]	Terminology was not understandable or did not match with the hard copy record	Equipment availability strongly affected staff perception (Number of workplaces and breakages)	Frustration set in when expectations were not met, problems not solved in a timely manner. This increased staff suspicion and decreased desire to work with the system.
	Did not match what they intended to chart	The lack of equipment failures, and PC availability were viewed as contributing to overtime work and led to distrust in the system	All levels of staff indicated it was difficult to maintain a positive attitude about the system and move forward when the implementation wasn’t going smoothly
	Staff appeared less comfortable without guidance	The lack of IT support, and PC availability were viewed as contributing to overtime work	When issues hindered job performance that led to dislike of the system and uncertainty about how to use the system correctly
	Initial and ongoing training was a prominent theme		
Cherry et al. [39]	Complex systems are difficult to navigate		Improved staff retention because of a sense of pride and empowerment associated with using computers in the work setting.
	Barriers frequently mentioned were the quality of staff training		
			Fear of computers were identified as a barrier
	Concerns about training		
	Strong initial and follow-up training		
Cherry et al. [40]	Learning to use the computer is a negative aspect	The primarily disadvantage consistently reported by all were related to technology problems and maintenance	Administrators reported that the system contributed employee satisfaction and staff retention (“*the facility is viewed as more modern and more attractive to potential new staff.”)*
			Nurses in supervisory positions were overwhelming positive about the system and would be very opposed to going back to the “precomputer” days.
			Direct Care Staff:
			Managers had a greater respect because they cared enough to give them computers for their work
Munyisia et al. [26]	Data in computerized records was located in various sections of the electronic system and, thus, difficult for the PCs and even the doctors to track the trend	Computerized documentation was not feasible at the bedside	
	*We want a chronological view of this data to make care decisions*		
	*I am able to go from one resident to the next using the electronic system, but when I have 35 residents, that is a lot of clicking and switching screens*		
	PCs charted certain information items on both paper and on a computer		
	*We report blood pressure data on paper because we get very frustrated looking for a resident’s data from various sections of the electronic system.*		
	Caregivers practice of double charting was partly caused by the way nursing data was organized in the system, making the data inconvenient to review		
Rantz et al. [41]	Entered data could not be located later	The primarily disadvantage related to technology problems and maintenance	Some view documentation as a “waste of time” and documentation takes time away from the residents
	Ongoing and refresher training of staff is important		
			Licensed and certified staff expressed concern that they could be watched by the monitoring of their documentation. On the other side, others saw the monitoring as a positive addition, since when reviewing the documentation they would know that the staff completed their assigned work Frustration set in when the system don’t work (that causes more time)
		Technology could be frustrating when it did not work	
	Using paper created a double documentation system. This creates more problems since information is inconsistently transferred		
Yu et al. [35]	Some felt the software was very easy to use		
	Some wished for more practice instead of lessons		
Zhang et al. [42]	*We are still learning I feel, we are learning something new every day*		

Italicized quotations represent the views of participants of included studies. Non-italicized quotations represent views of authors of included studies.

CNA = Certified Nurse Assistant.

DON = Director of Nursing.

PC = Personal Carer.

## Results

This review includes seven articles which summarize the findings of six studies. The articles of Alexander et al. [38] and Rantz et al. [41] report on the same study but synthesize different data. Three of the six studies (represented in four articles) were carried out in the U.S.A. [38-41] and three in Australia [26,35,42].

At least 320 semi-structured interviews and 56 focus groups were applied to form the database. The interviews were undertaken with management staff and direct care givers.

The 23 interviews compiled by Alexander et al. [38] are included in the 120 interviews with Rantz et al. [41]. Therefore they were subtracted from the remainder of the interviews. The same applies to the 22 focus groups in said articles by Alexander et al. [38] and Rantz et al. [41]. Observation data was compiled in three studies: The study by Cherry et al. [40] includes 10 one-hour observations. The study by Alexander et al. [38] and Rantz et al. [41] involve a series of observations, each consisting of a period of less than five minutes. Information regarding the frequency of observations was not made available in either of the two noted publications. From the study by Munyisia et al. [26] and Yu [35] only the qualitative findings were included in this review.

### Staff experiences within the implementation process

The key concepts of each article are shown in Tables 4 and 5. The following main interconnected themes arose from the analysis:

(1) Different information processing

(2) Quality of documentation and resident care needs

(3) Additional or lost time (1 – 3 shown in Table 4)

(4) Ease of use and ability to use it

(5) Equipment availability and technical functionality

(6) Attitude (4 – 6 shown in Table 5)

#### Different information processing

The implementation of IT requires a different form of information processing. Some individuals experience that as a benefit, describing easier access to charts and medical information, additional information, or a better and faster overview [26,38-42]. For those individuals, this leads to the examination of residents’ records without wasting time [39]. For example, they emphasize that missing charts no longer matter because the information is in the computer [40]. They also emphasize the benefit that computerized notes from all the caregivers are much easier to read than paper-based, handwritten records [40]. They appreciate being able to view many things about resident care at once and to know what is being done for their residents at that very moment [38,42]. They also appreciate that information on residents, including diagnosis and demographics, is now more readily available [26,40,41]. Leaders with the duty of guidance and control express their additional experience of being able to check what care has been supplied and of being able to monitor more easily the documentation of residents’ care activities, regulatory compliance issues, or staff education needs [39,40]. They also report that the IT facilitates their performance appraisal [42].

On the other hand, for some individuals IT is not experienced as a benefit. For various reasons they find it is more difficult to enter data. This leads to not entering data, to frustration and to using workarounds, e. g. double documentation with paper. For these employees, IT complicates their daily working processes [38,40-42].

#### Quality of documentation & resident care needs

Improvement in the quality of residents’ records leads to improvement in the quality of care because of more information and a broader and more holistic view of the residents. A quick response to resident’s care needs is possible, as are quicker and easier care decisions. Therefore the system has an impact on clinical judgment and decision-making [38-42].

More specifically, members of staff report that the system helps thinking and decision-making because of the ability to provide automatic alerts to help check plausibility [26,40-42]. This again leads to more consistent and legible documentation. In addition assessment templates guide through body systems for documentation and help improve observation skills [40]. Furthermore, the system causes the staff to think about what to assess and helps identify problems that might not be found otherwise [39,41,42]. Care feels safer through the use of the system [41].

In that case, the quality of residents’ records is lacking due to many circumstances, so that some residents do not receive the necessary care [40].

#### Additional or lost time

The question of time is a prominent theme within the implementation process. Some individuals experience a “given time” and benefit from the different information processing because of the IT, spending less time charting [26,39,40,42]. For those individuals who do not benefit from the different form of information processing it feels like “time is taken away” because of the system [26,30,38,41,42].

From a leader perspective, the outstanding expectation is that the IT always saves time for the staff when computer-based documentation is in force [41].

However, it does not matter how it is experienced: whether it feels as if time is given or lost. The time itself is always connected with what should actually have been done or what can be done in the future within this time, namely spending more time with the residents and giving better care. Given time, of course, is always experienced as a benefit.

#### Attitude

There are different views about using IT for documentation, ranging from feeling monitored to receiving greater respect. On the one hand, from a supervisory perspective, the monitoring of staff is viewed as a benefit. On the other hand, being monitored is experienced by the direct care staff both positively and negatively [41]. Some see it as a control, others view it differently: as accurately recording and recognizing tasks carried out and as a medium for greater respect [39,40]. Frustration has been reported when expectations were not met or problems were not solved in a timely manner. This increases staff suspicion and decreases the desire to work with the system [38]. These reported factors are summarized within the attitude to computers or to electronic systems. The attitude is either a hindering or promoting factor for experiencing benefits through the IT.

#### Ease of use & ability to use it

Some feel the software is easy to use and suitable for the daily working processes [35].

Others experience that the system’s terminology does not match what they intend to record [26,38] which led to double charting [26] or difficulty navigating [38]. For them, reviewing information is felt as inconvenient because of the way the data is organized in the electronic system. Besides, some could not locate entered data later [41]. That again causes job performance issues.

The ease of use must be seen in connection with the ability to use it. Learning to use the computer is of course a process as can be seen in dependence to Benner [44]. Without guidance staff appears less comfortable [38,41]. However, it is not only quantity that plays a role [39,40]. The training space and equipment is a fundamental factor. The ease of use and ability to use it is either a hindering or promoting factor for experiencing benefits through the IT.

#### Equipment availability and technical functionality

The difficulties encountered with a system collapse, due to technical problems, is always experienced as negative, time consuming and hindering. When such technical problems lead to overtime work or indifferent patient care, frustration follows. A lack of technology support, missing equipment (workplaces) and general maintenance issues increase the feeling of frustration and distrust. The equipment availability and technical functionality is either a hindering or promoting factor for experiencing benefits through the IT [26,38,40,41].

#### Line-of-argument synthesis

Our line-of-argument synthesis aimed at developing a model to explain staff experiences within the process of IT implementation. One construct resulted from our synthesis, namely: between benefit and burden.

Within the implementation process, the staff is always located between benefit and burden.

If the right promotion factors are available, the different information processing leads to an information value and is sensed as a benefit. This may lead to better quality of documentation and consequently to a better quality of care. The IT simplifies their daily working processes and for this reason connected with the feeling of given time. IT is sensed as a benefit.

If promotion factors are missing, the different information processing leads to information deficiencies and complicates the ability to fulfil the task. This may lead to poorer documentation, perhaps with time-consuming workarounds, and ultimately to a lower quality of care. In that case, IT complicates the daily working processes and is perceived as a burden.

The line between benefit and burden is semipermeable, depending on the impacting factors and may change at any time.

## Discussion

This study provides insights into staff experiences of benefits through the IT and the issues that hinder or promote the experiencing of benefits.

While IT-outcomes are extremely difficult and costly to measure [6-9], the method of asking the end-user seems to be an adequate and promising solution. As mentioned in the beginning of this study, Ammenwerth et al. [43] and Urquhart et al. [15] stated that quantitative methods might not be sufficient to explore why wards react differently to computer-based nursing documentation.

However, the implementation of an electronic documentation system does not lead automatically to a perceived benefit for the staff [3,25]. The staff is more likely to experience IT within the implementation process depending on the benefit gained through IT. Implementation strategies should address this consideration. Various factors affect experience and therefore the benefit. In principle, the impacting factors are known although the findings differ in sort and shape e.g. [4,11,18-20,22,24,28,29,33,39,43,45]. The known factors also appear in this underlying study but equipment availability and technical functionality is more prominent than in other studies. This allows the hypothesis that regulating this facilitating factor might suffice to receive a full information system success and could be a key to full computer system success.

Therefore, it is astonishing that none of the studies characterize the selection phase. With a specification sheet or a visiting reference, low technical functionality could be better controlled [3]. In respect of this selection phase, Alexander et al. and Rantz et al. [38,41] propose that staff should lower their expectations. But a different way to deal with this is to expand the planning phase and turn the attention from other specific preparations to a detailed system specification with fewer options for interpretation [3].

It is widely anticipated that the implementation of IT will reduce time on documentation but that must be differentiated. IT *could* minimize documentation time e.g. [5] but *must not* e.g. [25]. Many factors influence the outcome. It seems there is no linear increase. Instead the separation line is semipermeable. This means if an employee experiences a benefit in the form of reduced documentation time at point “A” that might change at point “B” due to modified influencing factors.

Studies thereby confirm that quality of care is directly related to quality of information [13,15]. But the quality of information is controlled by the outcome of the different information system and if this is expressed as a burden, care as well as satisfaction and the experience of benefits might be lacking. It is a vicious circle. It is therefore imperative to find out what is needed in order to simplify the daily working processes with IT and to satisfy staff.

So how much resource is necessary to ensure the simplification of their daily work? There is as yet no suggestion pointing to how much resource the management should invest and how much and what kind of resource is needed to definitely satisfy the staff and simplify their daily working processes. There is also as yet no suggestion as to how the change of resource during the project changes the satisfaction and, respectively, the sensed benefit through IT.

It is interesting to see that all the studies reviewed conclude that IT is worth the cost expenditure. The studies are generally positive about the benefits of IT even if the studies’ details represent another situation, namely staff reactions between benefit and burden.

To conclude, costs and benefits should be well balanced. Therefore RACFs should define the aims they are searching for with IT and relate these aims with the realistically reachable and possible benefits that staffs could experience. Finally, IT could benefit the daily working processes.

## Recommendations for further research

More qualitative research is needed to confirm the latter assumptions. Due to the fact that the reported experiences of nursing leaders were different to those of the direct, front-line nurses, more research is also needed on this topic. According to Patter, DeLone and McLean [46] more research is needed on the relationship between information quality and use, user satisfaction, and net benefits. Future studies should apply more comprehensive and consistent measures of usage in order to better understand the effect of the use of IT systems on user satisfaction and net benefits [46]: 258.

## Study limitations

Study limitations include the different settings in which these studies were conducted. This limits the ability to generalize the findings. Secondly, the timing of data collection was different in all studies, which could mean that experiences may differ.

## Conclusions

In summary, the implementation of an electronic documentation system does not lead automatically to a received benefit for the staff. Instead, the findings showed that within the implementation process the staff are always located between benefit and burden. Staff experience IT as a benefit when it simplifies their daily working routines. On the other hand, when IT complicates their daily working routines IT is experienced as a burden. Whether IT complicates or simplifies their routines depends on influencing factors. The edge between benefit and burden is semipermeable and may change at any time. The staff experience differs according to duties and responsibilities.

## Abbreviations

EHR: Electronic Health Record; IT: Information Technology; RACF: Residential Aged Care Facilities.

## Competing interests

The authors declare that they have no competing interests.

## Authors’ contributions

AM carried out the literature search, included and excluded documents, and wrote the manuscript. WS revised it critically for important intellectual content. All authors read and approved the final manuscript.

## Authors’ information

Anne Meißner has overseen the implementation of Electronic Nursing records in Long Term Care for many years. Due to her practical experience, her desire for deeper insight arose. She is undertaking her PhD on this topic in part-time study with no funding.

## Pre-publication history

The pre-publication history for this paper can be accessed here:

http://www.biomedcentral.com/1472-6947/14/54/prepub

## References

[B1] Ammenwerth E Haux R IT-Projektmanagement in Krankenhaus und Gesundheitswesen: Einführendes Lehrbuch und Projektleitfaden für das taktische Management von Informationssystemen 2005 Stuttgart: Schattauer

[B2] Cherry B Assessing organizational readiness for electronic health record adoption in long-term care facilities J Gerontol Nurs 2011 37 14 19 10.3928/00989134-20111102-01 21919421

[B3] Meißner A Althammer T Pflegedokumentation mit EDV: Richtig entscheiden, erfolgreich einführen 2012 Vincentz Network: Hannover

[B4] Fleischmann N Einstellungen und Haltungen von Pflegekräften gegenüber EDV-gestützter Dokumentation Pflegewissenschaft 2009 10 161 169

[B5] Lüngen M Gerber A Rupprecht C Lauterbach KW Effizienz der computergestützten Dokumentation in Pflegeheimen-eine Pilotstudie Pflege Z 2008 61 334 339 18605616

[B6] Kreidenweis H Halfar B IT-Report für die Sozialwirtschaft 2008/2009 2009 Eichstätt-Ingolstadt: Katholische Universität Eichstätt-Ingolstadt

[B7] Kreidenweis H Halfar B IT-Report für die Sozialwirtschaft 2010 2010 Eichstätt-Ingolstadt: Katholische Universität Eichstätt-Ingolstadt

[B8] Kreidenweis H Halfar B IT-Report für die Sozialwirtschaft 2011 2011 Eichstätt-Ingolstadt: Katholische Universität Eichstätt-Ingolstadt

[B9] Kreidenweis H Halfar B IT-Report für die Sozialwirtschaft 2012 2012 Eichstätt-Ingolstadt: Katholische Universität Eichstätt-Ingolstadt

[B10] Althammer T Sehlbach O Mehr schlecht als Recht Zum aktuellen Stand von Datenschutz und Datensicherheit in der Pflege und im Sozialwesen 2012. Ergebnisse einer Befragung von 295 Leitungskräften [01.06.2013: http://www.althammer-it.de/images/documents/Datenschutz-Studie-Pflege-2012.pdf]

[B11] Dyck MJ Nursing informatics. Applications for long-term care J Gerontol Nurs 2002 28 30 39 12382458 10.3928/0098-9134-20021001-10

[B12] Brandeis GH Hogan M Murphy M Murray S Electronic health record implementation in community nursing homes J Am Med Dir Assoc 2007 8 31 34 10.1016/j.jamda.2006.09.013 17210500

[B13] Häyrinen K Saranto K Nykänen P Definition, structure, content, use and impacts of electronic health records: A review of the literature Int J Med Inform 2008 77 291 304 10.1016/j.ijmedinf.2007.09.001 17951106

[B14] Munyisia EN Yu P Hailey D The changes in caregivers‘ perceptions about the quality of information and benefits of nursing documentation associated with the introduction of an electronic documentation system in a nursing home Int J Med Inform 2011 80 116 126 10.1016/j.ijmedinf.2010.10.011 21242104

[B15] Urquhart C Currell R Grant MJ Hardiker NR Nursing record systems: effects on nursing practice and healthcare outcomes Cochrane Database Syst Rev 2009 21 1 CD002099 19160206 10.1002/14651858.CD002099.pub2

[B16] DeLone W McLean E The DeLone and McLean model of information systems success. a ten-year update J Manag Inform Syst 2003 19 4 30

[B17] Ammenwerth E Iller C Mahler C IT-adoption and the interaction of task, technology and individuals: a fit framework and a case study BMC Med Inform Decis Mak 2006 6 10.1186/1472-6947-6-3 PMC1352353 16401336

[B18] Ammenwerth E Mansmann U Iller C Eichstädter R Factors affecting and affected by user acceptance of computer-based nursing documentation: results of a Two-year study J Am Med Inform Assoc 2002 10 69 84 12509358 10.1197/jamia.M1118 PMC150360

[B19] Carayon P Cartmill R Blosky MA Brown R Hackenberg M Hoonakker P Hundt AS Norfolk E Wetterneck TB Walker JM ICU nurses‘ acceptance of electronic health records J Am Med Inform Assoc 2011 18 812 819 10.1136/amiajnl-2010-000018 21697291 PMC3197984

[B20] Courtney K Alexander G Demiris G Information technology from novice to expert: implementation implications J Nurs Manag 2008 16 692 699 10.1111/j.1365-2834.2007.00829.x 18808463 PMC4389627

[B21] Daly J Buckwalter K Maas M Written and computerized care plans. organizational processes and effect on patient outcomes J Gerontol Nurs 2002 28 14 22 12240516 10.3928/0098-9134-20020901-05

[B22] Ehrenberg A Nurses perceptions concerning patient records in Swedish nursing homes Nurse Sci Quaterly 2011 24 9 14 10.1177/0894318410389065

[B23] Hakes B Whittington J Assessing the impact of an electronic medical record on nurse documentation time Comput Inform Nurs 2008 26 234 241 10.1097/01.NCN.0000304801.00628.ab 18600132

[B24] Lee T Nurses‘ concerns about using information systems: analysis of comments on a computerized nursing care plan system in Taiwan J Clin Nurs 2005 14 344 353 10.1111/j.1365-2702.2004.01060.x 15707445

[B25] Munyisia EN Yu P Hailey D Does the introduction of an electronic nursing documentation system in a nursing home reduce time on documentation for the nursing staff? Int J Med Inform 2011 80 782 792 10.1016/j.ijmedinf.2011.08.009 21956002

[B26] Munyisia EN Yu P Hailey D The impact of an electronic nursing documentation system on efficiency of documentation by caregivers in a residential aged care facility J Clin Nurs 2012 21 2940 2948 10.1111/j.1365-2702.2012.04157.x 22827170

[B27] Ossip-Klein DJ Karuza J Tweet A Howard J Obermiller-Powers M Howard L Katz P Griffin-Roth S Swift M Benchmarking implementation of a computerized system for long-term care Am J Med Qual 2002 17 94 102 10.1177/106286060201700304 12073871

[B28] Steffan S Laux H Wolf-Ostermann K Einstellungssache IT-gestützte Pflegedokumentation? Printernet 2007 14 94 101

[B29] Stevenson JE Nilsson G Nurses‘ perceptions of an electronic patient record from a patient safety perspective: a qualitative study J Adv Nurs 2012 68 667 676 10.1111/j.1365-2648.2011.05786.x 21781148

[B30] Stevenson J Nilsson G Petersson GI Johansson PE Nurses‘ experience of using electronic patient records in everyday practice in acute/inpatient ward settings: A literature review Health Informatics J 2010 16 63 72 10.1177/1460458209345901 20413414

[B31] de Vliegher K Paquay L Vernieuwe S van Gansbeke H The experience of home nurses with an electronic nursing health record Int Nurs Rev 2010 57 508 513 10.1111/j.1466-7657.2010.00827.x 21050204

[B32] Vogelsmeier A Halbesleben JRB Scott-Cawiezell JR Technology implementation and workarounds in the nursing home J Am Med Inform Assoc 2008 15 114 119 17947626 10.1197/jamia.M2378 PMC2274876

[B33] Whittaker A Aufdenkamp M Tinley S Barriers and facilitators to electronic documentation in a rural hospital J Nurs Scholarsh 2009 41 293 300 10.1111/j.1547-5069.2009.01278.x 19723278

[B34] Yeh S Jeng B Lin L Ho T Hsiao C Lee L Chen S Implementation and evaluation of a nursing process support system for long-term care: a Taiwanese study J Clin Nurs 2009 18 3089 3097 10.1111/j.1365-2702.2009.02879.x 19825114

[B35] Yu P Hailey D Li H Caregivers‘ acceptance of electronic documentation in nursing homes J Telemed Telecare 2008 14 261 265 10.1258/jtt.2008.080310 18633002

[B36] Noblit GW Hare RD Meta-ethnography: Synthesizing Qualitative Studies, Volume 11 1988 Newbury Park, Calif: Sage Publications

[B37] Moher D Liberati A Tetzlaff J Altman DG Preferred reporting items for systematic reviews and meta-analyses: the PRISMA statement BMJ 2009 339 b2535 10.1136/bmj.b2535 19622551 PMC2714657

[B38] Alexander GL Rantz M Flesner M Diekemper M Siem C Clinical information systems in nursing homes: an evaluation of initial implementation strategies Comput Inform Nurs 2007 25 189 197 10.1097/01.NCN.0000280589.28067.18 17625399

[B39] Cherry B Carter M Owen D Lockhart C Factors affecting electronic health record adoption in long-term care facilities J Healthc Qual 2008 30 37 47 18411891 10.1111/j.1945-1474.2008.tb01133.x

[B40] Cherry BJ Ford EW Peterson LT Experiences with electronic health records: early adopters in long-term care facilities Health Care Manage Rev 2011 36 265 274 10.1097/HMR.0b013e31820e110f 21646885

[B41] Rantz MJ Alexander G Galambos C Flesner MK Vogelsmeier A Hicks L Scott-Cawiezell J Zwygart-Stauffacher M Greenwald L The use of bedside electronic medical record to improve quality of care in nursing facilities: a qualitative analysis Comput Inform Nurs 2011 29 149 156 20975545 10.1097/NCN.0b013e3181f9db79

[B42] Zhang Y Yu P Shen J The benefits of introducing electronic health records in residential aged care facilities: a multiple case study Int J Med Inform 2012 81 690 704 10.1016/j.ijmedinf.2012.05.013 22749424

[B43] Ammenwerth E Mansmann U Mahler C Kandert M Eichstädter R Are quantitative methods sufficient to show why wards react differently to computer-based nursing documentation? Med Inform Eur 2001 2002 90 377 381 15460721

[B44] Benner PE From Novice to Expert: Excellence and Power in Clinical Nursing Practice 2001 Upper Saddle River, N.J: Prentice Hall

[B45] England I Stewart D Walker S Information technology adoption in health care: when organizations and technology collide Aust Health Rev 2000 23 176 185 10.1071/AH000176 11186051

[B46] Petter S DeLone W McLean E Measuring information systems success: models, dimensions, measures, and interrelationships Eur J Inform Syst 2008 17 236 263 10.1057/ejis.2008.15

